# Improv as cognitive activity

**DOI:** 10.3389/fnagi.2025.1520698

**Published:** 2025-03-20

**Authors:** Kristin R. Krueger, Jeffrey P. Winer, Daniel C. Lattimore, Todd Beck, Kyle Dennis, Cameron Carswell, Clifton Saper, Mathieu Hainselin

**Affiliations:** ^1^Rush Institute for Healthy Aging, Rush University Medical Center, Chicago, IL, United States; ^2^Boston Children’s Hospital and Harvard Medical School, Boston, MA, United States; ^3^Omiyo Consulting, LLC, Cincinnati, OH, United States; ^4^Private Practice, Chicago, IL, United States; ^5^Private Practice, Evanston, IL, United States; ^6^Department of Psychology, University of Picardie Jules Verne, Amiens, France

**Keywords:** cognitive activity, aging, healthy aging, attention, memory, improv theater exercises

## Abstract

**Background:**

Engaging in regular cognitive activity has been associated with cognitive function, yet the field of aging research has limited choices of cognitive activity programs to implement in clinical trials. As the field of aging research works to operationalize healthy habits, the potential role of improvisational theater (*improv*) to improve the lives of older adults has emerged. Given the limitations of existing cognitive training programs and the promise of improv, we sought to establish the feasibility of creating a cognitive training program based on improv exercises.

**Methods:**

We engaged 13 neuropsychologists and trainees in 15 improv exercises and asked them to rate the extent to which each exercise engaged or required one of 20 distinct cognitive abilities or cognitive subdomains. We then examined the mean ratings of the highest and the lowest rated subdomains to provide evidence that each exercise could be mapped onto different cognitive subdomains, thereby providing evidence of concept.

**Results:**

Our results demonstrated that these informed participants deemed the improv exercises as engaging cognitive processes. We found consensus among raters via higher-than-average means for specific abilities across the 15 exercises. Ratings from participants were broadly consistent with the pre-study groupings of the authors.

**Discussion:**

Our study provides the initial steps of establishing construct validity of improv exercises as a meaningful form of cognitive activity. This set of exercises can be examined as a cognitive training program in future clinical trials in order to determine if it has a significant influence on the cognitive function of older adults.

## Introduction

Engaging in regular cognitive activity has been identified as one of the modifiable risk factors for dementia reduction, yet the field of aging research has yet to establish specific recommendations for cognitive activity (Livingston et al., 2020). Epidemiological studies have established cognitive activity as an important lifestyle factor associated with cognitive function (Yates et al., 2016) despite remaining questions (Sajeev et al., 2016). In contrast, research on cognitive training (CT) programs in late life, have provided mixed evidence for the influence of cognitive training on cognitive function outcomes (Butler et al., 2018; Gates et al., 2019). This discrepancy in findings is likely influenced by multiple factors, such as study design and size, as well as the nature of the cognitive activities employed in the studies, which is the focus of this paper. Epidemiologic studies have typically identified a set of common leisure activities (e.g., reading) and then demonstrated the association with better cognitive function (Marquine et al., 2012; Wilson et al., 2012) and lower risk of cognitive decline (Krell-Roesch et al., 2019; Verghese et al., 2006) and dementia (Scarmeas et al., 2001; Wilson et al., 2021). In contrast, the majority of cognitive training programs have been designed to engage targeted areas of cognitive functioning (e.g., attention). With few exceptions (Noice and Noice, 2009; Noice et al., 2004), the tasks that make up the program are typically created specifically for cognitive training purposes (Ball et al., 2002) and may include strategies for improvement (Rebok et al., 2007). As programs developed in the laboratory – albeit based on principles of brain plasticity (Smith et al., 2009)– the activities are typically not common, familiar, or long-standing. Studies of CT programs have tended to look at the influence of specifically designed interventions on neuropsychological test performance (Hampshire et al., 2019; Rebok et al., 2007) and in some cases included everyday activities (Rebok et al., 2014; Willis et al., 2006). There has also been a great focus on the near transfer and far transfer of CT programs to neuropsychological test results (Gobet and Sala, 2023). In sum, epidemiological studies have tended to examine the association of real-world cognitive activities with cognitive decline and dysfunction (diagnoses that employ neuropsychological test data). CT programs have - by and large - studied the association of laboratory-developed tasks with neuropsychological test data.

Due to this noteworthy gap between the methods and results of epidemiological studies and CT programs, we reviewed the methods of epidemiological studies to develop a CT program, that had a basis in real-world activities, but that could be utilized in an intervention study context (e.g., clinical trial). We began with the early work on late-life cognitive activity by Wilson et al. (1999). Wilson et al. (1999) took seven basic activities (watching television, listening to the radio, reading a book, reading a magazine, reading a newspaper, playing games such as cards, checkers, crosswords, or other puzzles or games, and visiting a museum) that were determined to have “information processing central to it,” and had study participants rate the items on a 5-point scale regarding how frequently they engaged in them. For all of the activities, with the exception of visiting museums, participants were then asked to provide information on subtypes of activities, such as which section of the newspaper they read (Wilson et al., 1999). Wilson et al. (1999) also formed a panel of raters (10 doctoral psychologists and 20 lay persons) to estimate the level of cognitive intensity involved in each one of the seven basic activities and each subtype generated for the six basic activities (not visiting a museum). In the past 25 years, these seven basic activity items have been used in multiple studies and associated with better cognitive function, and slower rate of cognitive decline (Wilson et al., 2012) in diverse cohorts (Wilson et al., 1999; Marquine et al., 2012) and provided evidence against the reverse causation claim (Wilson et al., 2013; Wilson et al., 2021; Krueger et al., 2023).

As the field struggles to define the construct of cognitive activity as intervention, the theater arts, including improvisational theater, have shown promise in improving the well-being of older adults. Noice et al. (2004) planted the original seeds of promise that a structured theater arts program could improve the cognitive functioning of older adults in small, yet meaningful studies (Noice and Noice, 2009). Improv, as it is applied in the service of psychological constructs, draws upon the historical definitions of improv and is applied within the context of mental health intervention to address a specific psychological construct. The work of Viola Spolin is described as “each game is focused on a problem that the group of players should solve via playful experience (Sills and Sills, n.d.).” More recently, as mapped out by Keisari et al. (2024), small studies using improvisational training or *improv* have provided evidence for increased attentional abilities in older adults. Specifically, in a small study of older adults, 71–98 years old, engaging in an improv exercise, demonstrated improved attentional abilities compared to a movement-only control group (Keisari et al., 2022). Indeed, improvisational training has been applied in other settings to improve creative and divergent thinking (Felsman et al., 2020; Hainselin et al., 2018), but not yet in older adults on a large scale in research.

Given the lack of effective and efficacious CT programs and the promise of improv, we sought to reconcile the difference in methodology between epidemiological findings and targeted CT programs. Drawing upon the recommendations of Green et al. (2019), we describe a feasibility study that we conducted to test the primary hypothesis that improv exercises would be recognized as a form of cognitive activity by experts and trainees in the field of neuropsychology, and the secondary hypothesis that improv exercises can be codified and assembled to serve as a CT program. We modeled the process after Wilson et al. (1999) and engaged participants in improv exercises, thereafter asking them to rate each exercise on the cognitive skill they tapped. As such, we have begun the process of establishing construct validity for a CT program that can be used in future clinical trials with older adults.

## Materials and methods

### Participants

Participants were invited to participate in the study via neuropsychology listservs and email solicitations to clinical psychology training departments that had neuropsychology as a specialty. Participants were included if they identified as neuropsychologists or neuropsychologists in training. There were no exclusion criteria. Participants were given the option of coming to one of two sessions held in a conference room of the sponsoring hospital. They completed a one-page demographic sheet that queried, age, gender, race, and ethnicity, stage of training (e.g., board-certified neuropsychologist, trainee), number of years in practice, and improv/theater training experiences. All participants received three professional continuing education units (CEUs) and a gift card for $50 to help with transportation costs.

Nine neuropsychologists and four neuropsychologists-in-training participated in this study. Please see Table 1 for participant characteristics. The majority of participants were improv-naïve, with four participants (31%) indicating they had taken an improv class previously and two of those four endorsing other theater training.

**TABLE 1:** Participant characteristics.

Characteristic	*N* = 13^1^
**Age in years**	
Mean (SD)	42 (13)
(Range in years)	(26–62)
Female gender	8 (62%)
Non-Hispanic white	10 (77%)
Non-Hispanic black	1 (7.7%)
Non-Hispanic Asian	1 (7.7%)
Hispanic	1 (7.7%)
**Years practicing**	
Mean (SD)	8 (10)
(Range in years)	(0–30)
Board certified neuropsychologist	4 (31%)
Practicing neuropsychologist	5 (38%)
Neuropsychologist in training	4 (31%)

^1^Mean (SD); *n* (%).

### Improv exercises

In the broader theater arts and improv lexicon there are hundreds, if not thousands, of “games” that could be conceptualized as improv exercises. These exercises are based on philosophies, training techniques, and ideas described in noteworthy publications from leaders of modern-day improv (Spolin, 1999; Johnstone, 2007) and further elaborated in numerous publications in the last two decades (Levy, 2005; McGehee, 2007; Amador, 2018). We worked to select exercises that are commonly used in improv education and improv community performance settings. Authors (KRK, JW, who have a background in psychological assessment and improv) reviewed a wide range of short-form improv exercises that could be demonstrated in a group setting and conceivably be mapped onto five of the six neurocognitive domains, as defined in the Diagnostic and Statistical Manual of Mental Disorders, Fifth Edition (American Psychiatric and Association, 2013). We chose to use this framework of neurocognitive domains because the DSM-5 is widely used, comprehensive, and detailed. The DSM-5 (2013) defines six neurocognitive domains and several corresponding subdomains in relation to neurocognitive disorders. They are:

1. Complex attention (sustained attention, divided attention, selective attention, processing speed),
2. Executive function (planning, decision making, working memory, responding to feedback/error correction, overriding habits/inhibition, mental flexibility),
3. Learning and memory (immediate memory, recent memory, very-long-term memory, implicit learning),
4. Language (expressive, receptive),
5. Perceptual-motor (visual perception, visuospatial construction, perceptual-motor, praxis, gnosis), and
6. Social cognition (recognition of emotions, theory of mind).

We created a list of possible exercises and categorized them under one of the first five cognitive domains listed above. Since social cognition encompasses a large and important number of abilities and is often evaluated separately from the other more traditional cognitive domains, we chose to limit our study to the first five cognitive domains. Undoubtedly, the effect of improv on social cognition merits its own study.

Since there is variability in how improv exercises may be taught and carried out, we drafted descriptions of the exercises in order to standardize our understanding and ultimate execution of the exercise, as well as to allow others to reproduce this study. The lineup that was administered in the intervention was as follows: (1). Name game, (2). Red ball, (3). Wind-Rewind, (4). Zip Zap Zop (all with sliding clap), (5). Pass the look, (6). Buzz, (7). Receiving circle, (8). Picture description, (9), Object work introduction, (10). Zip, Zap, Zop (Blade, Blade, Clap), (11). Labeling, (12). Category Patterns, (13). Two-part words, (14). Mind Meld, and (15). Yeah/Boo. Please see Supplementary Table 1 for descriptions.

### Rating sheets

Participants were given a rating sheet with the 20 subdomains of cognitive abilities listed below each of the corresponding cognitive domain. For each exercise, participants rated each subdomain on a scale of 0 (not engaging the subdomain at all) to 6 engaging the subdomain in a full and complete manner). Participants were asked to rate the exercise immediately following their participation in the exercise to maximize their recollection of the details of the exercise. Participants had access to the DSM-5 descriptions of cognitive subdomains to reference as they conducted their ratings.

### Intervention

At each of the two sessions, we had the main interventionist (KRK) along with an assistant (CS, DL) who demonstrated the exercises and facilitated leading the participants in the exercises. Participants were instructed that they were serving as a panelist on a panel of experts who would rate each exercise on the cognitive ability tapped by the exercise. They were instructed to do so in an independent manner. Following the intervention, rating sheets were collected and participants engaged in a debriefing session. Participants were given a chance to reflect on the experience of engaging in improv exercises as a form of cognitive activity. The study was approved by the Institutional Review Board of the Cook County Health and Hospital System.

### Statistical analyses

We calculated descriptive statistics of the participant demographic characteristics. We also calculated the mean, standard deviation, and range for each of the cognitive abilities or subdomains, per improv exercise. We examined statistical consistency in ratings in several ways. First, we were interested in whether the participants would classify the individual exercises into domains in the same way as the authors. To this end, we used Cohen’s kappa, to estimate concordance between authors’ classification of exercises into domains and the participants’ classification of exercises into domains (based on subdomain ratings). Secondly, we were interested in the consistency of raters of the subdomains tapped by each exercise. To this end, we employed intraclass correlations (ICC) to examine the variability in rating attributable to the subdomain (versus rater) of each exercise. In other words, the intraclass correlation (ICC) by exercise measures the percent of the variability in rating attributable to the subdomain for each exercise. If most of the variability is due to the subdomain, as opposed to being due to the rater, there will be a high ICC or high consistency. Finally, we measured the consistency of raters of the exercise targeting each subdomain. We employed ICC to examine the variability in rating attributable to the exercise (versus rater) of each subdomain. If most of the variability is due to the exercise, as opposed to being due to the rater, there will be a high ICC or high consistency. Statistical analyses were conducted in R Core Team (2024).

## Results

### Participants

Qualitatively, during the debriefing session, participants overall expressed having enjoyed the exercises and experiencing positive feelings regarding taking a closer look at how cognitive activities correspond to cognitive domains.

### Ratings of improv exercises

First and foremost, we wanted to know if our participants would see the exercises as a form of cognitive activity, that is, exercises that use a high degree of information processing, as defined by Wilson et al. (1999), and were able to rate the exercises as tapping cognitive functions. Participants completed all items on all rating sheets and there were only two missing data points, presumably due to unintended omissions.

#### Activation of subdomains across exercises

##### Ratings indicating strong engagement of subdomains

For each exercise, we identified the cognitive ability or subdomain that received ratings with the highest mean. In Table 2, we list for each exercise the cognitive subdomain with the highest mean rating. We also provide standard deviation and range of ratings, as markers of the level of variability of ratings among participants. Following the highest mean is the ability with the second highest mean rating and then any rating that was 3.0 or greater. For a listing of all of the ratings, please refer to the Supplementary Table 2.

**TABLE 2:** Improv exercises and the corresponding cognitive ability with the highest mean ratings and the lowest mean ratings (shaded).

	Name game M (sd) range	Red ball M (sd) range	Wind-rewind M (sd) range	Zip Zap Zop all clap M (sd) range	Pass the look M (sd) range	Buzz M (sd) range
Sustained attention	3.8 (1.0) 2–5*	3.9 (1.1) 2–5	4.5 (1.1) 2–6	4.4 (1.0) 3–6	3.6 (1.2) 2–6	4.2 (1.2) 2–6
Divided attention	–	–	–	–	–	3.2 (2.0) 0–6
Selective attention	–	–	–	–	–	
Processing speed	4.1 (1.3) 1–6	3.2 (1.4) 1–5	–	4.1 (1.4) 2–6	–	3.2 (1.3) 1–6
Planning	–	–	–	–	–	–
Decision making	–	–	–	–	–	–
Working memory	–	–	4.0 (1.4) 2–6	–	–	4.3 (1.2) 2–6
Error correction	–	–	–	–	–	–
Inhibition	–	0.4 (0.5) 0–1	–	–	–	3.2 (1.8) 0–6
Mental flexibility	–	–	–	–	–	–
Immediate memory	4.2 (1.7) 0–6	3.6 (2.1) 0–6	5.0 (1.3) 2–6	3.2 (1.7) 1–6	–	–
Recent memory	–	–	4.1 (1.6) 0–6	–	–	–
Very long-term memory	0.1 (0.3) 0–1	0.08 (0.3) 0–1	0.2 (0.6) 0–2	0 (0) 0	0.1 (0.3) 0–1	–
Implicit learning	–	–	–	–	–	–
Expressive language	–	–	–	–	–	–
Receptive language	–	–	–	–	–	–
Visual-perception	–	–	–	–	–	–
Visuo-construction	0 (0) 0	–	0 (0) 0	0 (0) 0	0 (0) 0	0.1 (0.3) 0–1
Perceptual-motor	–	–	0.2 (0.6) 0–2	–	–	0.2 (0.4) 0–1
Gnosis	–	–	–	–	–	–

*The ability or subdomain with the highest mean rating, the second highest mean rating and all of the abilities with ratings of 3.0 and higher were listed in the table. The shaded cells represent the ability or subdomain with the lowest mean values.

##### Ratings indicating weak engagement of subdomains

**FIGURE 1:**
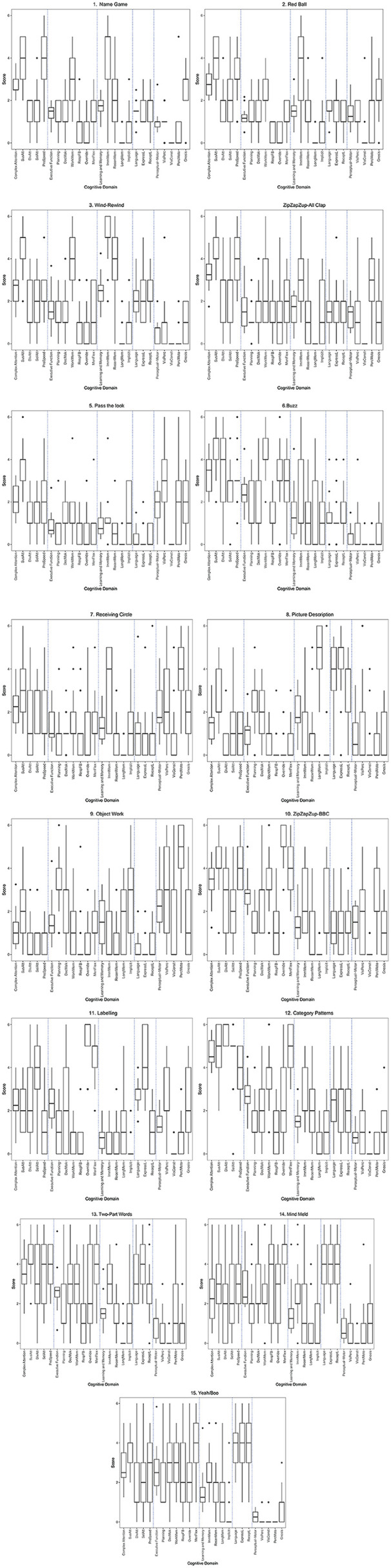
Improv exercises and cognitive domain ratings.

While one or more clear high means of 3.0+ emerged for each exercise, the remaining subdomains ranged from 0 to 2.9. A mean rating of 0 for any subdomain was rare but occurred for visual construction (*N* = 4), long-term memory (*N* = 1), and perceptual motor (*N* = 1). The variability of ratings is depicted in Figure 1, where the cognitive domain is listed, followed by the subdomains along the x-axis. The subdomain box plots are based on the participant ratings, while the domain box-plot is an average of the subdomain ratings.

#### Variability in ratings across exercises

Different patterns of ratings emerged for different exercises as demonstrated in Table 2. We can illustrate this finding with two exercises as examples. The Wind-Rewind game was rated by participants most highly in immediate memory (*M* = 5.09; sd = 1.3), followed by recent memory (*M* = 4.1; sd = 1.6) and working memory (*M* = 4.0, sd = 1.4), and having the lowest load on visuospatial construction (*M* = 0, sd = 0), followed by very long-term memory (*M* = 0.2, sd = 0.6) and perceptual motor (*M* = 0.2, sd = 0.6). In contrast the Picture Description exercise was rated highest by participants as engaging very long-term memory (*M* = 4.5; sd = 1.9), expressive language (*M* = 4.2; sd = 1.7) and receptive language (*M* = 3.1; sd = 1.6), and lowest on error correction (*M* = 0.3; sd = 0.9) and inhibition (*M* = 0.2; sd = 0.6.

In looking at the consistency of ratings, results were mixed. ICC values are most commonly interpreted as follows: less than 0.5 suggest poor reliability, values between 0.5 and 0.75 suggest moderate reliability, values between 0.75 and 0.9 suggest good reliability, and values greater than 0.90 suggest excellent reliability (Koo and Li, 2016). In our study, we found approximately 50% of the consistency of ratings of exercises were good, the other half were poor. Approximately one-third of the ratings of subdomains were good and the rest were poor. See Table 3 for ICC for exercises and subdomains.

**TABLE 3:** Intraclass correlations (ICC) among exercise ratings and domain ratings.

Exercise	ICC	Domain	ICC
Name game	0.6	DivAtt	0.47
Red ball	0.48	SelAtt	0.37
Wind-rewind	0.69	ProSpeed	0.51
Zip Zap Zup (all clap)	0.48	Planning	0.32
Pass the look	0.41	DecMak	0.25
Buzz	0.49	WorkMem	0.52
Receiving circle	0.32	RespFB	0.31
Picture description	0.5	Override	0.67
Object work	0.46	MenFlex	0.7
Zip Zap Zup (BBC)	0.54	ImmMem	0.42
Labeling	0.58	RecenMem	0.33
Category patterns	0.65	LongMem	0.56
Two-part words	0.48	Implicit	0.16
Mind meld	0.44	ExpressL	0.64
Yeah/boo	0.53	ReceptL	0.47
	–	VisPerc	0.27
	–	VisConst	0.18
	–	PercMoto	0.53
	–	Gnosis	0.18
	–	DivAtt	0.47

#### Concordance between authors’ ratings and participants’ ratings

Participant ratings of the main cognitive domain tapped in the improv exercises were generally consistent with the author’s original conception of the exercise. Participants’ ratings of 12 of the 15 exercises, based on the highest mean, matched the authors’ ratings. The discordantly rated exercises were Category Patterns, Two-part Words, and Mind Meld. In one of these discordantly rated exercises (Mind Meld), the second highest domain rating from participants matched the author’s original rating. Overall, the estimated concordance between participant and author ratings, using Cohen’s kappa was 0.769, an indication of a “moderate” concordance (McHugh, 2012).

### Discussion

We engaged neuropsychologists and neuropsychologists-in-training in 2.5 h of improv exercises and found that all of the participants regarded the improv exercises as a form of cognitive activity that engaged several specific cognitive abilities. While there was considerable variability in ratings among the subdomains, participant ratings were overall consistent with the authors’ original conceptualization at the domain level. This paper is the first to assess the cognitive processes involved in improv exercises with expert raters.

We found robust evidence for our primary hypothesis as participants rated all of the exercises as engaging multiple cognitive abilities. We established consensus of ratings by examining the highest and the lowest means for each subdomain within each exercise. We found that within each exercise high means, low means, and mid-means emerged, providing evidence that participants perceived individual exercises to engage certain subdomains of cognitive abilities to a high degree, and other subdomains, to a low or null degree. Mid-means also emerged in some exercises, suggesting that the exercises tapped several subdomains simultaneously to a variable degree. As measured by ICC, consistency of ratings was mixed, with only 50% of the exercise ratings and 33% of subdomain ratings receiving designations of “good.” There may be several reasons for this finding. Low consensus of the cognitive load at the subdomain level may have been due to our inability to adequately convey the rating task to all of our participants. Alternatively, the mixed ratings may be due to the complex nature of human activities – that simultaneously tap multiple domains. Or perhaps the mixed ICC findings lie in the lack of consensus in the field as to ecological validity of cognitive domains/subdomains. Attempting to obtain concordance in ratings of activities that tap specific cognitive ability was ambitious and is a process that requires further refinement in future studies. In fact, there is little to no information available on this type of concordance from other CT programs. Conversely, the fact that the improv exercises potentially engaged multiple domains simultaneously may be an indicator that improv is a rich source of cognitive activity. The acknowledgment of improv as a cognitive activity holds, despite our inability to have a high level of agreement on the intensity of information processing for specific cognitive subdomains.

A noteworthy finding is that 11 out of 15 of the exercises were rated as having a higher-than-average mean in sustained attention. Sustained attention was defined as “Maintenance of attention over time (e.g., pressing a button every time a tone is heard, and over a period of time).” (American Psychiatric and Association, 2013). Sustained attention is a cognitive ability that is essential to daily living and susceptible to changes as we age (Staub et al., 2013), and in turn may affect other cognitive abilities (Vallesi et al., 2021). Furthermore, deficits in sustained attention may be able to differentiate individuals without dementia from those with dementia (Manuel et al., 2019). In this sense, a CT program loads heavily on sustained attention may have a positive impact on sustained attention, and in turn on other cognitive abilities. As is the nature of improv, certain exercises are often learned and then initially executed slowly, until proficiency builds. As soon as there are signs of proficiency of an exercise, the speed is increased. This is believed to be a technique that aids actors on stage “think on their feet” and learn to respond quickly. It is no surprise, then that processing speed was the cognitive subdomain that had the second number of high means (*N* = 8). Similarly, this bodes well for the establishment of a CT program, as slowed processing speed is one of the cognitive abilities that is most often found in normal aging (Wilson et al., 2002). On the other hand, participants found that the exercises did not tap visuospatial construction abilities or at best minimally. In summary, the improv exercises were rated as covering a wide range of cognitive abilities with a high demand on sustained attention and processing speed, but low requirements for visuospatial construction abilities.

Similar to Wilson et al. (1999), we chose activities that were practiced on a regular basis by people for reasons other than cognitive health, but that were hypothesized to be related to cognitive health. We differ from Wilson et al. (1999) in that improv exercises are typically practiced in the context of theater arts, which requires specialized training. Like Wilson et al. (1999), we surveyed expert panelists regarding the items of interest in a standardized manner so that we would be able to reproduce our proposed intervention program.

Our approach differs from computerized cognitive training (CCT) approach that creates computer exercises to target specific areas of cognitive functioning. According to the website of BrainHQ, one of the widely applied CCT programs, Michael Mezernich, PhD (Mezernich, 2023) describes how his team developed Brain HQ’s exercises, “There are several different elemental skills, or abilities, or dimensions of your operations that need to be refined. And there’s a relatively large literature in psychophysics — the study of human perception and recognition — that we draw on. Then, we create tasks that apply these rules of brain plasticity so that we can efficiently drive improvements in the machinery of the brain.” In contrast to these goals of CCT development, our goal was to explore the cognitive activity potential of a rich theater art, which could also address social and psychological outcomes simultaneously. Improv exercises are largely conducted in supportive group settings, in contrast to CCT programs that are designed to be completed individually. It might be because of this supportive group format that improv exercises lend themselves to help create human bonding (Keisari et al., 2024). It is also important to acknowledge that engaging in improv exercises has few side effects for the individuals, although occasional frustration may be one potential side effect. When frustration does arise, it can be leveraged into a training opportunity. Improv exercises can be conducted in many environments without any special technology and with different numbers of participants, from 1 to 20+, with an ideal number between 6 and 12 persons. The exercises can be adapted, scaled, and delivered in diverse intervention contexts including school and outpatient-based mental health settings (Ellis et al., 2020) or inpatient/partial hospital settings (Winer et al., 2018). In general, the exercises in our study can be adapted for different cognitive capacities, by adjusting the speed of instruction, number of practice trials, and content of suggestions. For example, for an exercise that involves more physical - compared to verbal – action, such as “Red Ball” the instructions can be explained and practiced as many times as necessary. Having a savvy assistant helps to convey the exercises for groups of persons with variable cognitive abilities. Improvisers who lead are adept at shifting expectations to meet the group’s dynamic, so ideally this is a built-in quality of the execution of any session. For exercises that are designed to generate new verbal ideas, such as “Category Patterns,” the suggestions may vary based on group. In groups of older adults, where some cognitive decline is known or suspected (e.g., preclinical and early mild cognitive impairment), requesting suggestions related to the immediate environment (e.g., reasons to come to a library) or personal preference are desired (e.g., your favorite dessert). In groups of adults for whom a high level of cognitive ability is more certain, suggestions can be bolder (e.g., their favorite character in a fiction book). While this study has a focus on cognitive training for persons without a diagnosis of dementia, we realize that many groups of older adults may include a range of cognitive abilities. Future work in this area may benefit from familiarity with the distinct, but related work using improv with persons with dementia (Zeisel et al., 2018) and using improv to enhance the experience of caregivers for persons with dementia (Kemp et al., 2024).

Considering theater art exercises in the context of cognitive and affective health is not without precedence. Noice et al. (2004) found that after a 4 weeks theater training intervention community-dwelling older adults demonstrated significantly better cognitive function in recall and problem-solving, and enhanced psychological well-being compared to the no-treatment control group. In a second study, Noice and Noice (2009) found similar cognitive and emotional gains in a group of older adults living in subsidized retirement homes. Noice and Noice (2009) points out that their acting intervention differs from the other short-term training programs in that their training does not directly target performance on the test measures, suggesting that a less direct training program may provide answers to the field. Indeed, there may be additional reasons to employ improv exercises with older adults since they provide a highly social context. This aspect is increasingly important, at a time when social isolation and loneliness are considered epidemic in the United States (Office of the Surgeon General, 2023). Furthermore, we have reason to believe that improv interventions may reduce symptoms of depression and anxiety (Krueger et al., 2017), although this has not yet been studied in cohorts of older adults.

Our study has several strengths. We were able to explore a target (improv exercises) for a CT program that examined activities that are practiced for reasons other than dementia prevention while outlining a system for these activities to be studied in clinical trials. We provide evidence for a list of improv exercises that may be considered cognitive activities and can be further studied. We had raters consider subdomains of cognitive abilities, as we know that subdomains even within the same cognitive domain may tap disparate abilities. Therefore, a summary measure of subdomains (e.g., a domain) may lack meaning in this context. The substance of our program, improv exercises, are vibrant, interesting, and naturally structured in such a way that participants engage readily in them. Our study may also help to identify the appropriate skills needed to train health professionals who work with older adults (Chan et al., 2023). More widely, this study could help future improv research to choose the exercises to include in their workshop programs.

Our study also has limitations. We used one conceptualization of cognitive abilities or subdomains (American Psychiatric and Association, 2013), that is comprehensive and widely used; however, there may be other conceptualizations that lend themselves to this task. For example, we might have been able to simplify the process by only rating domains used in research on aging (e.g., episodic memory, semantic memory, perceptual speed, verbal fluency, working memory and visual spatial ability, Krueger et al., 2009) and future iterations may consider this. Although we engaged participants in a wide range of improv exercises, exercises that engage visuospatial constructional abilities were lacking. Our results are limited to the feasibility of this concept and to fully understand the potential of improv exercises as a CT program, this will need to be tested in a randomized clinical trial, with a larger number of participants, in future research. This paper is a first step toward better identification of cognitive processes involved in improv. We hope it will help future research groups and improv facilitators to build their programs and assess them accordingly.

### Conclusion and future directions

Improv exercises were regarded as cognitive activities, activities that engaged multiple cognitive domains by informed participants. As such, improv exercises hold promise for the field of brain health among older adults and these results should encourage future studies that improve upon the fidelity of this study. For instance, specific time limits on individual exercises should be defined; domains/subdomains should be pruned and more attention should be given to training raters. Intervention studies, including larger clinical trials, are needed to evaluate if cognitive function improves following participation in an improv-based cognitive activity intervention, determine optimal dosing, and evaluate what gains, if any, are maintained over time.

## Data Availability

The raw data supporting the conclusions of this article will be made available by the authors, without undue reservation.

## References

[B1] Amador S. (2018). *Teaching Social Skills Through Sketch Comedy and Improv Games.* Philadelphia: Jessica Kingsley Publisher.

[B2] American Psychiatric and Association. (2013). *Diagnostic and Statistical Manual of Mental Disorders: DSM-5*, 5th Edn. Washington, DC: American Psychiatric Publishing. 10.1176/appi.books.9780890425596

[B3] Ball K. Berch D. B. Helmers K. F. Jobe J. B. Leveck M. D. Marsiske M. (2002). Advanced cognitive training for independent and vital elderly study group. Effects of cognitive training interventions with older adults: A randomized controlled trial. *JAMA* 288 2271–2281. 10.1001/jama.288.18.2271 12425704 PMC2916176

[B4] Butler M. McCreedy E. Nelson V. A. Desai P. Ratner E. Fink H. A. (2018). Does cognitive training prevent cognitive decline? A systematic review. *Ann. Int. Med.* 168 63–68. 10.7326/M17-1531 29255842

[B5] Chan C. A. Chou E. LaDisa A. G. Mehta A. Zelenski A. Longtin K. (2023). Using nominal group technique to determine skills that applied improvisation can teach health profession education learners. *PEC Innov.* 3:100194. 10.1016/j.pecinn.2023.100194 37576803 PMC10415759

[B6] Ellis B. H. Abdi S. M. Winer J. P. (2020). Mental health practice with immigrant and refugee youth: A socioecological framework. *Am. Psychol. Assoc.* 10.1037/0000163-000

[B7] Felsman P. Gunawardena S. Seifert C. M. (2020). Improv experience promotes divergent thinking, uncertainty tolerance, and affective well-being. *Think. Skills Creativity* 35:100632. 10.1016/j.tsc.2020.100632

[B8] Gates N. J. Rutjes A. W. Di Nisio M. Karim S. Chong L. Y. March E. (2019). Computerised cognitive training for maintaining cognitive function in cognitively healthy people in late life. *Cochrane Database Syst. Rev.* 3:CD012277. 10.1002/14651858.CD012277 30864187 PMC6414816

[B9] Gobet F. Sala G. (2023). Cognitive training: A field in search of a phenomenon. *Perspect. Psychol. Sci.* 18 125–141. 10.1177/17456916221091830 35939827 PMC9903001

[B10] Green C. S. Bavelier D. Kramer A. F. Vinogradov S. Ansorge U. Ball K. K. (2019). Improving methodological standards in behavioral interventions for cognitive enhancement. *J. Cogn. Enhancement* 3 2–29. 10.1007/s41465-018-0115-y

[B11] Hainselin M. Aubry A. Bourdin B. (2018). Improving teenagers’ divergent thinking with improvisational theater. *Front. Psychol.* 9:1759. 10.3389/fpsyg.2018.01759 30319485 PMC6167459

[B12] Hampshire A. Sandrone S. Hellyer P. J. (2019). A large-scale, cross-sectional investigation into the efficacy of brain training. *Front. Hum. Neurosci.* 13:221. 10.3389/fnhum.2019.00221 31338032 PMC6629869

[B13] Johnstone K. (2007). *Impro. Performance Books.* London: Methuen Drama.

[B14] Keisari S. Feniger-Schaal R. Palgi Y. Golland Y. Gesser-Edelsburg A. Ben-David B. (2022). Synchrony in old age: Playing the mirror game improves cognitive performance. *Clin. Gerontol.* 45 312–326. 10.1080/07317115.2020.1799131 32762289

[B15] Keisari S. Krueger K. R. Ben-David B. M. Hainselin M. (2024). New horizon in improving ageing with improvisational theatre. *Age Ageing* 53:afae087. 10.1093/ageing/afae087 38706392

[B16] Kemp C. L. Craft Morgan J. Bender A. A. Hill A. M. Anglin E. Burgess E. O. (2024). “Just join them”: Improv and dementia care. *J. Appl. Gerontol.* 43 302–309. 10.1177/07334648231203195 37933156 PMC11267974

[B17] Koo T. K. Li M. Y. (2016). A guideline of selecting and reporting intraclass correlation coefficients for reliability research. *J. Chiropractic Med.* 15 155–163. 10.1016/j.jcm.2016.02.012 27330520 PMC4913118

[B18] Krell-Roesch J. Syrjanen J. A. Vassilaki M. Machulda M. M. Mielke M. M. Knopman D. S. (2019). Quantity and quality of mental activities and the risk of incident mild cognitive impairment. *Neurology* 93 e548–e558. 10.1212/WNL.0000000000007897 31292224 PMC6710000

[B19] Krueger K. R. Desai P. Beck T. Wilson R. S. Evans D. Rajan K. B. (2023). Cognitive activity is associated with cognitive function over time in a diverse group of older adults, independent of baseline biomarkers. *Neuroepidemiology* 57 229–237. 10.1159/000531208 37263261 PMC10997141

[B20] Krueger K. R. Murphy J. W. Bink A. B. (2017). Thera-prov: A pilot study of improv used to treat anxiety and depression. *J. Mental Health* 28 621–626. 10.1080/09638237.2017.1340629 28675707

[B21] Krueger K. R. Wilson R. S. Kamenetsky J. M. Barnes L. L. Bienias J. L. Bennett D. A. (2009). Social engagement and cognitive function in old age. *Exp. Aging Res.* 35 45–60. 10.1080/03610730802545028 19173101 PMC2758920

[B22] Levy G. (2005). *112 Acting Games.* Colorado Springs: Meriwether Publishing Ltd.

[B23] Livingston G. Huntley J. Sommerlad A. Ames D. Ballard C. Banerjee S. (2020). Dementia prevention, intervention, and care: 2020 report of the lancet commission. *Lancet* 396 413–446. 10.1016/S0140-6736(20)30367-6 32738937 PMC7392084

[B24] Manuel A. L. Foxe D. Bradshaw N. Cordato N. J. Hodges J. R. Burrell J. R. (2019). Sustained attention failures on a 3-min reaction time task is a sensitive marker of dementia. *J. Neurol.* 266 1323–1331. 10.1007/s00415-019-09261-9 30834482

[B25] Marquine M. J. Segawa E. Wilson R. S. Bennett D. A. Barnes L. L. (2012). Association between cognitive activity and cognitive function in older Hispanics. *J. Int. Neuropsychol. Soc.* 18 1041–1051. 10.1017/S135561771200080X 22676914 PMC3515684

[B26] McGehee L. (2007). *Plays Well With Others.* Austin: Dalton Publishing.

[B27] McHugh M. L. (2012). Interrater reliability: The kappa statistic. *Biochem. Med.* 22 276–282. 10.11613/BM.2012.031 PMC3900052 23092060

[B28] Mezernich M. (2023). *9 Burning Questions for BrainHQ’s Founder, Dr. Michael Merzenich.* Available online at: https://www.brainhq.com/better-brain-health/article/brain-training/9-burning-questions-brainhqs-founder-dr-michael-merzenich (accessed September 19. 2024).

[B29] Noice H. Noice T. (2009). An arts intervention for older adults living in subsidized retirement homes. *Aging Neuropsychol. Cogn.* 16 56–79. 10.1080/13825580802233400 18686051 PMC2769921

[B30] Noice H. Noice T. Staines G. (2004). A short-term intervention to enhance cognitive and affective functioning in older adults. *J. Aging Health* 16 562–585. 10.1177/0898264304265819 15271270

[B31] Office of the Surgeon General. (2023). *Our Epidemic of Loneliness and Isolation: The US Surgeon General’s Advisory on the Healing Effects of social Connection and Community.* Washington, DC: US Department of Health and Human Services. 37792968

[B32] R Core Team (2024). *R: A Language and Environment for Statistical Computing.* Vienna: R Foundation for Statistical Computing.

[B33] Rebok G. W. Ball K. Guey L. T. Jones R. N. Kim H. Y. King J. W. (2014). Ten-year effects of the advanced cognitive training for independent and vital elderly cognitive training trial on cognition and everyday functioning in older adults. *J. Am. Geriatrics Soc.* 62 16–24. 10.1111/jgs.12607 24417410 PMC4055506

[B34] Rebok G. W. Carlson M. C. Langbaum J. B. (2007). Training and maintaining memory abilities in healthy older adults: Traditional and novel approaches. *J. Gerontol. Ser. B Psychol. Sci. Soc. Sci.* 62 53–61. 10.1093/geronb/62.special_issue_1.53 17565165

[B35] Sajeev G. Weuve J. Jackson J. W. VanderWeele T. J. Bennett D. A. Grodstein F. (2016). Late-life cognitive activity and dementia: A systematic review and bias analysis. *Epidemiology* 27 732–742. 10.1097/EDE.0000000000000513 27227783 PMC5460628

[B36] Scarmeas N. Levy G. Tang M. X. Manly J. Stern Y. (2001). Influence of leisure activity on the incidence of Alzheimer’s disease. *Neurology* 57 2236–2242. 10.1212/WNL.57.12.2236 11756603 PMC3025284

[B37] Sills A. Sills C. no date. *Viola Spolin Biography, Viola Spolin.* Available online at: https://www.violaspolin.org/bio (accessed October 29, 2024).

[B38] Smith G. E. Housen P. Yaffe K. Ruff R. Kennison R. F. Mahncke H. W. (2009). A cognitive training program based on principles of brain plasticity: Results from the Improvement in memory with plasticity-based adaptive cognitive training (IMPACT) Study. *J. Am. Geriatrics Soc.* 57 594–603. 10.1111/j.1532-5415.2008.02167.x 19220558 PMC4169294

[B39] Spolin V. (1999). *Improvisation for the Theater: A Handbook of Teaching and Directing Techniques.* Evanston, IL: Northwestern University Press.

[B40] Staub B. Doignon-Camus N. Després O. Bonnefond A. (2013). Sustained attention in the elderly: What do we know and what does it tell us about cognitive aging? *Ageing Res. Rev.* 12 459–468. 10.1016/j.arr.2012.12.001 23261761

[B41] Vallesi A. Tronelli V. Lomi F. Pezzetta R. (2021). Age differences in sustained attention tasks: A meta-analysis. *Psychon. Bull. Rev.* 28 1755–1775. 10.3758/s13423-021-01908-x 33772477 PMC8642381

[B42] Verghese J. LeValley A. Derby C. Kuslansky G. Katz M. Hall C. (2006). Leisure activities and the risk of amnestic mild cognitive impairment in the elderly. *Neurology* 66 821–827. 10.1212/01.wnl.0000202520.68987.48 16467493 PMC1415273

[B43] Willis S. L. Tennstedt S. L. Marsiske M. Ball K. Elias J. Koepke K. M. (2006). Long-term effects of cognitive training on everyday functional outcomes in older adults. *JAMA* 296 2805–2814. 10.1001/jama.296.23.2805 17179457 PMC2910591

[B44] Wilson R. S. Beckett L. A. Barnes L. L. Schneider J. A. Bach J. Evans D. A. (2002). Individual differences in rates of change in cognitive abilities of older persons. *Psychol. Aging* 17:179. 10.1037//0882-7974.17.2.179 12061405

[B45] Wilson R. S. Bennett D. A. Beckett L. A. Morris M. C. Gilley D. W. Bienias J. L. (1999). Cognitive activity in older persons from a geographically defined population. *J. Gerontol. Ser. B Psychol. Sci. Soc. Sci.* 54 155–160. 10.1093/geronb/54B.3.P155 10363036

[B46] Wilson R. S. Boyle P. A. Yu L. Barnes L. L. Schneider J. A. Bennett D. A. (2013). Life-span cognitive activity, neuropathologic burden, and cognitive aging. *Neurology* 81 314–321. 10.1212/WNL.0b013e31829c5e8a 23825173 PMC3772831

[B47] Wilson R. S. Segawa E. Boyle P. A. Bennett D. A. (2012). Influence of late-life cognitive activity on cognitive health. *Neurology* 78 1123–1129. 10.1212/WNL.0b013e31824f8c03 22491864 PMC3320053

[B48] Wilson R. S. Wang T. Yu L. Grodstein F. Bennett D. A. Boyle P. A. (2021). Cognitive activity and onset age of incident Alzheimer disease dementia. *Neurology* 97 e922–e929. 10.1212/WNL.0000000000012388 34261788 PMC8408511

[B49] Winer J. P. Wadsworth L. P. Forgeard M. Pinder-Amaker S. Björgvinsson T. Beard C. (2018). Development and implementation of a single-session diversity and multicultural psychology group intervention within an academic psychiatric hospital. *Behav. Therapist* 41 327–334.

[B50] Yates L. Ziser S. Spector A. Orrell M. (2016). Cognitive leisure activities and future risk of cognitive impairment and dementia: Systematic review and meta-analysis. *Int. Psychogeriatrics* 28 1791–1806. 10.1017/S1041610216001137 27502691

[B51] Zeisel J. Skrajner M. J. Zeisel E. B. Wilson M. N. Gage C. (2018). Scripted-IMPROV: Interactive improvisational drama with persons with dementia-effects on engagement, affect, depression, and quality of life. *Am. J. Alzheimer’s Dis. Other Dement.* 33 232–241. 10.1177/1533317518755994 29504407 PMC10852482

