# Supplementary material for: Improv as cognitive activity
Source: Front Aging Neurosci. 2025 Mar 20;17:1520698. doi: 10.3389/fnagi.2025.1520698 (PMC11965582; doi:10.3389/fnagi.2025.1520698)
Supplement: Supplementary file 1 [file Table_1.docx]

CogniProv: Improv Exercises as Cognitive Activity

Jeff Winer and Kristin Krueger

Cognitive domains are based on the American Psychiatric Association DSM-5 descriptions (2013). American Psychiatric Association. (2013). *Diagnostic and
 statistical manual of mental disorders* (5th ed.). Washington, DC:
 Author.

Text citation: (American Psychiatric Association, 2013)

Selected improv exercises in the order they were presented to participants in the workshop:

1. Name Game
2. Red Ball
3. Wind-Rewind
4. ZipZapZop (all clap)
5. Pass the Look
6. Buzz
7. Receiving Circle
8. Picture Description
9. Object Work
10. ZipZapZop (BBC)
11. Labelling
12. Category Patterns
13. Two-part Words
14. Mind Meld
15. Yeah/Boo

The descriptions below are to be used for two main purposes: 1. Trained improvisers around the world may read them and understand the rules of the exercise. Perhaps they will recognize it as one they know with or without variations. For readers who are not improvisers, the description will convey a general idea of how improv exercises work from a leader’s position. This description does not substitute for training in improv because there are several principles that are learned in classes and in the practice of improv over time that are not contained in these descriptions.

**Complex Attention** (sustained attention, divided attention, selective attention, processing speed).

1. *Red Ball*: Players stand or sit in a circle. One player starts off, motioning their hands as if they had an imaginary red ball. They make eye contact with another player, call out their name (e.g. “Taylor, red ball!”), and mimic throwing the ball to said person. The receiver of the ball (Taylor), responds, “Thank you, red ball.” And repeats this action to another player. Clear eye contact with between person throwing the ball and person whose name has been called and will receive the ball, is important. The size/shape and weight of the ball, as indicated by the physical movements of players, can vary.
2. *Zip Zap Zop – All clap:* Players stand in a circle. The three words always follow the same sequence, starting with Zip. Each word is accompanied by the right hand slide-clapping into the left and then pointing in the direction of the person to whom the Zip is being passed. The person that receives the Zip then chooses another person, makes eye contact with them and then passes the focus with the same hand clap, saying Zap. This person passes the focus again to another person, using the same hand-slide clap and says Zop. The sequence begins again, with eye contact to a new person, clap and word. The exercise should start out slowly, until a couple of rounds go by, involving all players. Then the pace builds, making it more and more challenging.
3. *Pass the Look:* Players stand in a circle. The person who starts, makes eye contact with another and with an intentional purposeful look “passes the look.” The person who receives the look then passes it to another person.

**Executive Function**

1. *Zip Zap Zop – BBC:* This version of Zip Zap Zop is played in the same manner as the ZZZ version above, but with different hand movements. On Zip, the movement is simply the right hand extending and pointing in the direction of the person with whom they are also making eye contact. The right hand starts out like it is being extended for a hand shake but instead is sharp and points to the person of interest like a “blade.” Repeat this move for Zap. The person who passes on Zop, will clap. The hand movement pattern is blade, blade, clap or: Zip (blade), Zap (blade), Zop (clap).
2. *Labelling:* Have participants walk around the room, pointing at various objects in the room and calling out what they are. For example, “chair,” “piano,” “garbage can,” wall.” Do this for 3 minutes. Then ask the participants to walk around the room and point to an object, but say *what it is not*. For example, point to a chair and call it an “elephant.”
3. *Buzz*: The players can sit in a circle as they will count out loud, one by one around the circle. They will simply count 1, 2, 3, etc. except when there is a 7 in the number or a multiple of 7. Instead of saying the number (that contains 7 or is a multiple of 7), they say “Buzz” and the counting continues. If an error is made, the next person begins at number 1. It may sounds something like this: 1..2..3..4..5..6..Buzz..8..9..10..11..12..13..Buzz..15..16..Buzz..18.
4. *Yeah/Boo*: Two players sit next to each other slightly facing in. The rest of the players form an audience facing the two players. One player is assigned to be YEAH and one player is assigned to be BOO. YEAH always has a positive twist on the previous sentence. BOO always has a negative twist on the previous sentence. YEAH begins the exercise, usually with a sentence from the leader or audience, but more experienced players may come up with their own. The sentence should be in first person for YEAH. BOO will add a negative twist sentence in second person. The goal for YEAH is to find the positive in anything and everything. The goal for BOO is to find the negative in anything and everything. Immediately after YEAH’s sentence, the audience says in unison a light-hearted, upbeat: “Yeaaaaaah!” Immediately after BOO’s sentence, the audience says in unison, a sad, downbeat: “Booooooo.” The example provided to the participants can be something like:

YEAH: I just got a new job!

BOO: Right, but now you have to spend money on a new wardrobe.

YEAH: That’s great because I love spending money.

BOO: But then you will spend all your money on clothes and not have money to go to the movies. You’ll have to watch netflix instead.

YEAH: That’s perfect because I love watching movies after everyone else has seen them.

BOO: All the movies will be ruined because people will tell you what happens at the end before you see it.

YEAH: I’m so happy that movies will be ruined for me because my goal will be to spend more time having training for a road race.

BOO: If you start to train for a road race, you will get shin splints.

**Learning and Memory**

1. *Name Game:* Players stand in a circle. Each player, one after the other, states their name loud and clear so that everyone can hear. Each player is asked to pay attention so they can learn the names. If necessary, have the players repeat their names again. Once everyone has at least a couple of the names memorized, the exercise can begin. One person begins by making eye contact with another player and then says the players name, thereby passing the focus. This goes on until every player has taken 2-3 turns. Players are told that if they cannot think of a person’s name, they can ask the person their name, then say it and continue.
2. *Wind-*Rewind: Pick a category, such as “Favorite foods.” Going around the circle, each player says a favorite food (Wind). After everyone has taken their turn, the leader says, “All together) and then together in unison and in reverse order, everyone repeats the foods. The goal is for everyone to say the food, so each person should pay attention and if they cannot remember, they would look for cues or wait for the initial sound of the food so they can join in. It might look like this: First round – wind up: apples, pizza, sushi, hummus, tacos. Rewind together: Taaacos, huuumus, suuushi, piiiza, aaaples. When the words are drawn out, everyone gets a chance to join in.
3. *Category Patterns:* Pick one category, such as types of “breakfast cereals”, or “makes of cars.” Everyone raises one hand. If the category was breakfast cereals, the leader says a type of cereal while looking at a person of their choice. The leader keeps the hand raised because they will be the last person to complete the cycle. After the focus is passed by the leader, the person who has the focus makes eye contact with another person and says a different breakfast cereal and lowers their hand. This goes on until everyone has said a breakfast cereal and it comes back to the leader. A pattern has been established. Now the group repeats the pattern exactly as it was established. Practice this 2-3 times until the pattern has been well-learned. Then introduce the second category and establish a different pattern using the same process as was used to establish the first one. Practice this pater until it is well-learned, then you simultaneously begin the first pattern.

**Language**

1. *Two-part Words:* Players stand in a circle. The first person says a word, starting out with a simple word, such as ‘house.’ The following person adds a word to create a compound word building off of the previous suggestion, such as ‘boat.’ Then everyone says this word together, “houseboat” adding, da da da da at the end. Then the person who provided the additional word (‘boat’ in this example, provides a new root word. A rhythm is created, such that everyone slowly taps their legs and claps their hands and the first word is said on one beat. The second word is said on the second beat and then the compound is said on the third beat.
2. *Mind Meld:* Players stand in a circle. Two players jump in, say “1, 2, 3” and each says a random word that comes to their minds. Then together they say “1, 2, 3.” And say another word association based off of those two words that were stated. The ultimate goal is for the two players to state, at the same time the same word, through an iterative process. An example of how this might work. Both players: “1, 2, 3.” Player 1: “Viking.” Both players: “1, 2, 3.” Player 2: “Pineapple” --- Then both players think of a potential link…they count 1, 2, 3, and say together: “Spikes.” Players can be tagged out if someone in the circle has an idea for an association.
3. *Describe the Picture*: The leader gives everyone a blank piece of paper – usually an 8.5 x 11 with no lines. The task is for everyone to describe a picture from their life that they remember fondly – a picture of a happy event they can recall. Describe it to the group in such detail that others can imagine the scene and the feelings unfolding on the paper. [Also primary domain affected: Learning and Memory – because it taps autobiographical, long-term memory.]

**Perceptual-Motor**

1. *Receiving Circle*: Players stand in a circle. One person makes a gesture and sound at the same time – that they pass to this next person. The person who received the gesture/sound faces the next person in the circle and tries to imitate the gesture/sound – that the person passes to them, as they pass it to them - exactly. This exercise can go 2-3 rounds.
2. *Object Work*: Improvisers use their bodies to convey objects in lieu of using props. In this exercise, the leader demonstrates 10 common objects that are conveyed via object work.
