# Supplementary material for: Improv as cognitive activity
Source: Front Aging Neurosci. 2025 Mar 20;17:1520698. doi: 10.3389/fnagi.2025.1520698 (PMC11965582; doi:10.3389/fnagi.2025.1520698)
Supplement: Supplementary file 2 [file Table_2.docx]

Supplementary Table. Improv exercises and the corresponding cognitive ability with all means

|  | **Name game**  M (sd) range | **Red ball**  M (sd) range | **Wind-Rewind**  M (sd) range | **Zip Zap Zop**  **All clap**  M (sd) range | **Pass the look**  M (sd) range | **Buzz**  M (sd) range |
| --- | --- | --- | --- | --- | --- | --- |
| Sustained Attention | 3.8 | 3.9 (1.1) 2-5 | 4.5 (1.1) 2-6 | 4.4 (1.0) 3-6 | 3.6 (1.2) 2-6 | 4.2 (1.2) 2-6 |
| Divided Attention | 1.4 | 1.9 | 1.7 | 2.2 | 1.2 | 3.2 (2.0) 0-6 |
| Selective Attention | 1.7 | 2.1 | 1.9 | 2.3 | 1.7 | 2.8 |
| Processing Speed | 4.1 | 3.2 (1.4) 1-5 | 2.5 | 4.1 (1.4) 2-6 | 1.9 | 3.2 (1.3) 1-6 |
| Planning | 1.2 | 1.2 | 1.3 | 1.1 | 0.8 | 1.7 |
| Decision Making | 1.5 | 1.6 | 1.5 | 1.9 | 1.3 | 1.8 |
| Working Memory | 2.9 | 2.5 | 4.0 (1.4) 2-6 | 2.6 | 1.4 | 4.3 (1.2) 2-6 |
| Error Correction | 0.9 | 0.6 | 0.9 | 1.3 | 0.7 | 1.2 |
| Inhibition | 0.5 | 0.4 (0.5) 0-1 | 0.7 | 1.4 | 0.6 | 3.2 (1.8) 0-6 |
| Mental Flexibility | 1.4 | 1.4 | 1.5 | 2.2 | 0.9 | 2.6 |
| Immediate Memory | 4.2 (1.7) 0-6 | 3.6 (2.1) 0-6 | 5.0 (1.3) 2-6 | 3.2 (1.7) 1-6 | 1.4 | 2.3 |
| Recent Memory | 2.0 | 1.6 | 4.1 (1.6) 0-6 | 1.5 | 0.7 | 1.2 |
| Very Long-Term Memory | 0.1 (0.3) 0-1 | 0.08 (0.3) 0-1 | 0.2 (0.6) 0-2 | 0 (0) 0 | 0.1 (0.3) 0-1 | 1.4 |
| Implicit Learning | 0.9 | 1.0 | 0.9 | 1.5 | 1.4 | 1.1 |
| Expressive language | 1.5 | 1.6 | 2.2 | 1.7 | 0.3 | 1.3 |
| Receptive Language | 1.5 | 1.4 | 2.3 | 1.6 | 0.3 | 1.5 |
| Visual-Perception | 1.0 | 1.2 | 1.0 | 1.3 | 2.9 | 0.7 |
| Visuo-Construction | 0 (0) 0 | 0.4 | 0 (0) 0 | 0 (0) 0 | 0 (0) 0 | 0.1 (0.3) 0-1 |
| Perceptual-Motor | 0.6 | 1.8 | 0.2 (0.6) 0-2 | 2.8 | 2.2 | 0.2 (0.4) 0-1 |
| Gnosis | 2.5 | 2.3 | 1.1 | 1.2 | 2.0 | 0.7 |

|  | **Receiving Circle**  M (sd) range | **Picture Description**  M (sd) range | **Object work**  M (sd) range | **Zip Zap Zop**  **(BBC)**  M (sd) range | **Labelling**  M (sd) range | **Category Patterns**  M (sd) range |
| --- | --- | --- | --- | --- | --- | --- |
| Sustained Attention | 3.4 (1.5) 1-6 | 2.8 | 2.2 | 4.1 (1.3) 1-6 | 2.2 | 4.8 (1.4) 2-6 |
| Divided Attention | 1.7 | 0.5 | 0.7 | 2.8 | 2.7 | 5.6 (0.5) 5-6 |
| Selective Attention | 2.4 | 1.2 | 1.0 | 2.2 | 3.6 (1.8) 0-6 | 4.5 (1.7) 0-6 |
| Processing Speed | 1.7 | 1.0 | 0.8 | 4.1 (1.0) 3-6 | 1.2 | 3.8 (1.1) 2-5 |
| Planning | 1.0 | 2.5 | 3.3 (1.4) 1-6 | 1.4 | 1.2 | 1.5 |
| Decision Making | 1.1 | 1.9 | 2.4 | 2.8 | 2.3 | 2.2 |
| Working Memory | 1.8 | 0.9 | 1.2 | 3.4 (1.4) 1-6 | 0.9 | 3.7 (1.5) 1-6 |
| Error Correction | 1.2 | 0.3 (0.9) 0-3 | 0.6 | 1.3 | 0.5 | 1.3 |
| Inhibition | 1.2 | 0.2 (0.6) 0-2 | 0.6 | 4.8 (1.4) 1-6 | 5.1 (1.3) 3-6 | 2.6 |
| Mental Flexibility | 1.7 | 0.7 | 1.3 | 4.2 (1.2) 2-6 | 4.5 (1.2) 2-6 | 4.8 (1.2) 3-6 |
| Immediate Memory | 3.1 (1.8) 1-5 | 1.2 | 1.0 | 2.4 | 0.6 | 2.7 |
| Recent Memory | 1.0 | 1.2 | 0.7 | 1.1 | 0.7 | 1.9 |
| Very Long-Term Memory | 0.10 (0.3) 0-1 | 4.5 (1.9) 0-6 | 1.9 | 0.2 (0.4) 0-1 | 1.3 | 0.4 (0.7) 0-2 |
| Implicit Learning | 1.4 | 0.8 | 2.4 | 2.1 | 0.3 (0.6) 0-2 | 1.1 |
| Expressive Language | 1.0 | 4.2 (1.7) 0-6 | 0.2 (0.6) 0-2 | 1.2 | 4.5 (1.5) 2-6 | 2.5 |
| Receptive Language | 1.1 | 3.1 (1.6) 0-5 | 0.5 (0.8) 0-2 | 1.5 | 1.2 | 2.1 |
| Visual-Perception | 2.5 | 1.6 | 2.7 | 1.6 | 2.8 | 1.2 |
| Visuo-Construction | 0.9 (1.6) 0-5 | 0.6 | 1.4 | 0.2 (0.4) 0-1 | 0.1 (0.3) 0-1 | 0.1 (0.3) 0-1 |
| Perceptual-Motor | 3.9 (1.7) 1-6 | 0.4 | 4.3 (1.7) 1-6 | 2.8. | 0.8 | 0.5 |
| Gnosis | 2.0 | 0.9 | 1.5 | 1.2 | 2.2 | 1.5 |

|  | **Two-Part Words**  M (sd) range | **Mind Meld**  M (sd) range | **Yeah/Boo**  M (sd) range |
| --- | --- | --- | --- |
| Sustained Attention | 4.4 (1.2) 2-6 | 2.9 | 3.8 (0.9) 2-5 |
| Divided Attention | 3.0 (1.9) 0-6 | 2.0 | 2.0 |
| Selective Attention | 3.5 (1.6) 1-6 | 2.1 | 2.5 |
| Processing Speed | 3.8 (1.0) 2-5 | 3.1 (1.5) 1-6 | 2.6 |
| Planning | 1.8 | 1.9 | 2.1 |
| Decision Making | 2.9 | 2.8 | 3.0 (1.6) 0-6 |
| Working Memory | 3.0 (1.4) 1-5 | 2.4 | 2.9 |
| Error Correction | 1.6 | 3.2 (2.2) 0-6 | 2.4 |
| Inhibition | 2.9 | 2.6 | 2.2 |
| Mental Flexibility | 3.9 (1.4) 1-6 | 4.5 (1.1) 3-6 | 3.9 (1.3) 1-6 |
| Immediate Memory | 3.2 (1.1) 2-5 | 2.6 | 2.5 |
| Recent Memory | 1.7 | 1.7 | 1.8 |
| Very Long-Term Memory | 0.3 (0.6) 0-2 | 0.9 | 1.2 |
| Implicit Learning | 1.4 | 1.2 | 0.5 |
| Expressive Language | 3.8 (1.4) 2-6 | 3.8 (1.0) 2-5 | 3.9 (1.2) 1-6 |
| Receptive Language | 2.9 | 3.8 (1.6) 1-6 | 3.9 (1.4) 1-6 |
| Visual-Perception | 0.8 | 0.8 (1) 0-3 | 0.2 |
| Visuo-Construction | 0.2 (0.4) 0-1 | 0.1 (0.3) 0-1 | 0.1 (0.3) 0-1 |
| Perceptual-Motor | 1.9 | 0.8 | 0 (0) 0 |
| Gnosis | 1.0 | 1.1 | 0.9 |
